# Association between geriatric nutritional risk index and osteoarthritis in aged person over 60: data from NHANES 2005-2018

**DOI:** 10.3389/fmed.2025.1579095

**Published:** 2025-05-08

**Authors:** Hongxu Lu, Ping Chen, Yaozong Song, Yila Su, Wulan Tai, Baoleri Xilin

**Affiliations:** Department of Orthopedic, International Mongolia Hospital of Inner Mongolia, Inner Mongolia Autonomous Region, Hohhot, China

**Keywords:** geriatric nutritional risk index, osteoarthritis, risk factors, elderly, NHANES

## Abstract

**Background:**

Osteoarthritis (OA), a prevalent age-related degenerative joint disorder, demonstrates significant associations with nutritional status. This study examines the prognostic value of the Geriatric Nutritional Risk Index (GNRI) in OA risk stratification among elderly individuals.

**Methods:**

This retrospective analysis utilized seven NHANES cycles (2005-2018) encompassing geriatric participants (≥ 60 years) possessing complete GNRI measurements and baseline covariates. For comparative cohort balancing, propensity score matching was executed using inverse probability weighting a matched-pairs design, adjusting for age, alcohol consumption, and the Poverty Income Ratio. Multivariable-adjusted weighted logistic regression quantified GNRI-OA associations, with restricted cubic splines (RCS) characterizing nonlinear dynamics. Subgroup analyses were also performed.

**Results:**

This cross-sectional analysis identified 656 OA cases among 3,120 rigorously screened geriatric participants. Elevated GNRI levels demonstrated a significant association with increased OA risk among geriatric populations, with the correlation remaining robust in sensitivity analyses adjusted for metabolic confounders. Specifically, a GNRI ≥ 123.63 was associated with a higher probability of OA in this population. RCS analysis revealed a significant non-linear relationship (p_non-linear < 0.001) between GNRI and OA risk, particularly among men and non-smokers. Subgroup analyses indicated that males, Hispanic Americans, Non-Hispanic Black people, non-smokers, and those with a low PIR were more sensitive to changes in GNRI.

**Conclusion:**

Elevated GNRI was independently associated with OA prevalence in geriatric populations, demonstrating nutritional status’s pivotal role in degenerative joint pathophysiology. The impact of GNRI on OA risk may differ across demographic subgroups, highlighting the need for personalized approaches in managing OA risk based on nutritional status.

## 1 Introduction

Osteoarthritis (OA), a prevalent chronic condition, is marked by the progressive breakdown of articular cartilage and subchondral bone, leading to symptoms such as joint pain and reduced mobility, most frequently affecting the knee, hip, and hand joints. This condition ranks among the primary contributors to impaired mobility and functional limitations in the elderly population. Globally, the prevalence of OA is significant, affecting more than 7% of the global population (1). In the United States, over 30 million adults are affected by OA, with knee OA being particularly prevalent (2). This imposes a considerable burden on both individuals and society. Despite the high prevalence of OA, current therapeutic strategies focus on symptom control, such as pain relief and physiotherapy, with no definitive cure available other than joint replacement (3). This underscores the urgent need for further research into modifiable risk factors and potential preventive measures.

First proposed in 2005, the Geriatric Nutritional Risk Index (GNRI) (4), has emerged as a validated metric quantifying malnutrition-related risks in aging populations, incorporating serum albumin, and anthropometric parameters. Substantial evidence has demonstrated its clinical associations with multiple health outcomes including depression (5), osteoporosis (6), and cancer progression (7). Although low GNRI levels have been consistently associated with impaired bone health outcomes, in particular decreased bone mineral density in the hip, increased risk of fracture, and osteoporosis development (6, 8, 9). But evidence suggests that elevated GNRI may indicate over nutrition-related diseases, with recent studies linking higher scores to non-alcoholic fatty liver disease (10). This association underscores the rationale for our study, which identified a previously unrecognized link between elevated GNRI levels and increased OA risk in older adults.

Person-level factors related to OA, as reported in previous research, include serum 25-hydroxyvitamin D (11), smoking (12), lipid accumulation products (13), skeletal muscle index (14), systemic inflammatory response index (15), obesity, and BMD among others (16). Emerging evidence suggests that micronutrient profiles serve as modifiable determinants of OA. Analyses revealed that pro-inflammatory dietary patterns accelerate radiographic knee degeneration (17), while deficiencies in omega-3 fatty acids exacerbate inflammation (18). Another study emphasized that elevated body mass index (BMI) and obesity, frequently associated with suboptimal nutritional profiles, serve as key contributors to the pathogenesis of OA (19). Furthermore, inflammation, which is influenced by dietary components, has been implicated in the pathogenesis of OA (20). These findings suggest that nutritional interventions could potentially modify the risk and progression of OA.

Although the interplay between the GNRI and OA pathogenesis merits further investigation, this association remains poorly characterized in older populations. To address this gap, we analyzed data from the NHANES to specifically examine the predictive value of GNRI for OA occurrence among older U.S. adults. Our findings provide epidemiological evidence supporting the implementation of nutrition-focused clinical protocols and community-based prevention initiatives aimed at reducing OA-related disability in aging populations.

## 2 Materials and methods

### 2.1 Data preprocessing

The NHANES employs a population-based stratified sampling design administered by the National Center for Health Statistics (NCHS) under the sponsorship of the Centers for Disease Control and Prevention (CDC). This surveillance system methodically gathers comprehensive health and nutritional metrics from adult participants through standardized physical examinations and structured interviews. For this study, de-identified records were obtained covering the 2005–2018 survey periods, encompassing seven complete data collection cycles.

### 2.2 Definition of osteoarthritis in elderly individuals aged 60 and older

#### 2.2.1 Definition of osteoarthritis

OA patients were identified based on medical condition files (MCQ) in the NHANES survey. Participants were asked, “Has a doctor or other healthcare professional ever told you that you have arthritis?” Those answering “yes” were classified as arthritis patients. If the response was “no,” they were considered non-arthritis patients. Participants who responded “refused,” “don’t know,” or had missing data were excluded. Subsequently, arthritis patients were asked, “What type of arthritis do you have?” Those answering “osteoarthritis” were classified as osteoarthritis patients. Responses such as “rheumatoid arthritis” or “other” were considered non-osteoarthritis. Participants who refused, didn’t know, or had missing answers were excluded. Thus, the final cohort of arthritis patients was categorized as those with osteoarthritis (21).

#### 2.2.2 Definition of elderly individuals aged 60 and older

Elderly individuals were identified based on the demographic file (DEMO) in the NHANES survey, with those aged ≥ 60 years classified as elderly individuals (22).

### 2.3 GNRI

The formula for calculating GNRI is: GNRI = [1.489 × albumin (g/L)] + [41.7 × (current weight/ideal weight)], where ideal weight = 22 × height (m)^2^. When actual body weight exceeds ideal body weight, the weight ratio is truncated to 1.0. GNRI nutritional risk levels are defined as: nutritional risk (GNRI < 98) and adequate nutritional status (GNRI ≥ 98) (4).

**FIGURE 1:**
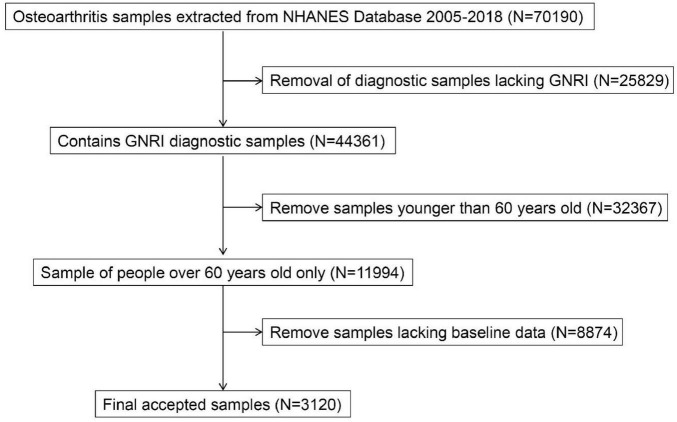
Flow chart.

From the preliminary dataset of 70,196 screened individuals, exclusion criteria encompassed: absence of GNRI diagnostic samples (*N* = 25,829), participants younger than 60 years (*N* = 32,367), and absence of baseline data (*N* = 8,874). The final study included 3,120 participants, of whom 656 were OA patients and 2,464 were healthy controls (Figure 1).

### 2.4 Covariates

Based on previous literature, several potential confounding factors were analyzed, including sex, age, ethnicity, height, weight, BMI, smoking, alcohol consumption, education, family income poverty ratio, albumin, lymphocytes, creatinine, total cholesterol, serum glucose, triglycerides, and monocyte count. Sex, age, ethnicity, education, and family poverty income ratio (PIR) were determined using the DEMO in the NHANES survey. Ethnicity was classified into the following groups: Mexican American, non-Hispanic Black people, non-Hispanic White people, other Hispanic, and other/multiracial. Education level was divided into five categories: less than 9th grade, 9–11th grade, high school graduate, associate degree, and college graduate or higher. Family income was categorized based on PIR: low income (< 4.99) and middle-high income ( ≥ 5). Alcohol consumption was determined by survey questions regarding the number of alcoholic drinks consumed in a given year. Those responding “1-36” drinks were classified as drinkers (DRINK). Laboratory data were obtained from the NHANES laboratory dataset.

### 2.5 Propensity score matching

PSM is a statistical method used to handle observational study data. In observational studies, bias and confounding variables often arise, and PSM is employed to reduce their impact, thereby enabling more accurate comparisons between experimental and control groups. This method was first introduced by Paul Rosenbaum and Donald Rubin in 1983 and is widely used in fields like medicine and public health to match baseline characteristics (23). To ensure comparability and similar distribution of baseline data between the OA group and the healthy elderly group, and to minimize the influence of baseline data on study results, a 1:1 propensity score matching was performed in this study based on age, alcohol consumption, and PIR. Post-PSM, both groups were analyzed to ensure the robustness and accuracy of the study results.

### 2.6 Weighted logistic regression analysis

Weighted binary logistic regression was employed to assess the potential relationship between GNRI and OA in individuals aged ≥ 60 years. GNRI was incorporated into the model as both a continuous and categorical variable, enabling the calculation of odds ratios (OR) and their 95% confidence intervals (95% CI). When GNRI was treated as a continuous variable, the original GNRI was divided into quartiles, arranged from low to high. These quartiles were labeled as the first quartile (Q1), second quartile (Q2), third quartile (Q3), and fourth quartile (Q4), and were further evaluated as categorical variables. The lowest GNRI quartile (Q1) served as the reference. We implemented three progressively adjusted analytical frameworks: (1) Model 1 without covariate control; Model 2 incorporating biological sex and racial/ethnic composition; and Model 3 additionally adjusted for alcohol consumption, lymphocyte count, creatinine, total cholesterol, triglycerides, and monocyte count. All regression analyses incorporated survey weights, and continuous covariates displaying non-normal distributions were transformed using weighted quartile adjustments. Interaction analyses were performed to evaluate potential subgroup-specific effects of GNRI. *P*-values were adjusted for false discovery rate.

### 2.7 Subgroup analysis

Subgroup analysis evaluated whether the relationship between GNRI and OA in elderly individuals aged 60 and older differed across various subgroups. This study also performed interaction effect analysis to assess whether there were differences in associations between GNRI and sex, ethnicity, smoking, education, and family PIR. Forest plots were used to visually compare the effect sizes and confidence intervals (CI) of multiple study results, providing a more intuitive understanding of the differences in odds ratios (OR) and their 95%CI between the two groups, and helping to better understand the consistency and variability across different studies.

### 2.8 Restricted cubic spline

RCS represents a statistical methodology frequently employed in regression analysis and curve fitting. This approach entails dividing the data range into distinct segments, within which a cubic polynomial is fitted to produce a smooth and continuous curve, thereby facilitating the modeling of continuous variables. These polynomials are smoothly connected across adjacent intervals, with additional constraints on smoothness to avoid sharp fluctuations in the curve. In statistical modeling, RCS are frequently used to model the relationship between continuous variables and the dependent variable, allowing for the capture of non-linear relationships while maintaining smoothness and avoiding overfitting.

### 2.9 Statistical analysis

Analytical workflows were executed in the R statistical environment (version 4.4.2). Demographic and clinical profiles were stratified by GNR1 quartiles using distribution-appropriate metrics: Gaussian-distributed parameters as mean ± SD, skewed measurements as interquartile ranges, and categorical traits as frequency distributions (n, %). To evaluate differences in variable characteristics across GNR1 quartiles, the Wilcoxon rank-sum test was employed for continuous variables and the Rao-Scott chi-square test for categorical variables. All inferential frameworks adhered to frequentist principles, incorporating two-tailed hypothesis tests with a significance threshold of *p* < 0.05.

## 3 Results

### 3.1 Baseline characteristics of the samples

A total of 3,120 samples were included in this study to compare the baseline characteristics of individuals aged 60 and above with respect to gender, age, race, height, weight, BMI, smoking, alcohol consumption, and the PIR, in relation to the incidence of OA. Prior to PSM, there were meaningful differences between the OA group and the healthy control group in individuals aged 60 and above regarding age, alcohol consumption, education level, and PIR (*P* < 0.001, *P* < 0.001, *P* < 0.001, respectively). However, no significant differences were observed after PSM (*P* = 0.44, *P* = 0.16, *P* = 0.55). Other baseline characteristics exhibited differences between the OA and healthy control groups both before and after PSM, including gender (PSM before *P* < 0.001, PSM after *P* < 0.001), race (PSM before *P* < 0.001, PSM after *P* < 0.001), height (PSM before *P* < 0.001, PSM after *P* < 0.001), BMI (PSM before *P* < 0.001, PSM after *P* < 0.001), smoking (PSM before *P* < 0.001, PSM after *P* = 0.001), education (PSM before *P* < 0.001, PSM after *P* = 0.014), albumin (PSM before *P* = 0.013, PSM after *P* = 0.005), creatinine (PSM before *P* < 0.001, PSM after *P* = 0.001), and GNRI (PSM before *P* = 0.006, PSM after *P* = 0.003). Following propensity score matching, covariate equilibrium was maintained across residual baseline measures encompassing weight, lymphocyte count, total cholesterol, serum glucose, triglycerides, and monocyte count, between the OA and healthy control groups before and after PSM. These findings suggest that these indicators were relatively balanced between the two groups of individuals aged 60 and above, and their potential confounding effects were controlled for in the comparisons (Table 1).

**TABLE 1:** Baseline Characteristics of OA in the NHANES (2005-2018).

Variable	*N*	Overall	Before PSM		*P*-value*^2^*	*N*	Overall	After PSM		*P*-value*^2^*
		***N* = 3,120^1^**	**OA**	**Health**			***N* = 1,312^1^**	**OA**	**Health**	
			***N* = 656^1^**	***N* = 2,464^1^**				***N* = 656^1^**	***N* = 656^1^**	
Gender	3,120				<0.001	1,312				<0.001
Male		2,052 (66%)	330 (50%)	1,722 (70%)			791 (60%)	330 (50%)	461 (70%)	
Female		1,068 (34%)	326 (50%)	742 (30%)			521 (40%)	326 (50%)	195 (30%)	
Age (years)	3,120	69 (7)	70 (7)	69 (7)	<0.001	1,312	70 (7)	70 (7)	70 (7)	0.44
Race	3,120				<0.001	1,312				<0.001
Hispanic American		328 (11%)	47 (7.2%)	281 (11%)			102 (7.8%)	47 (7.2%)	55 (8.4%)	
Other Hispanic		249 (8.0%)	29 (4.4%)	220 (8.9%)			86 (6.6%)	29 (4.4%)	57 (8.7%)	
Non-Hispanic White		1,774 (57%)	463 (71%)	1,311 (53%)			850 (65%)	463 (71%)	387 (59%)	
Non-Hispanic Black		609 (20%)	80 (12%)	529 (21%)			212 (16%)	80 (12%)	132 (20%)	
Other races		160 (5.1%)	37 (5.6%)	123 (5.0%)			62 (4.7%)	37 (5.6%)	25 (3.8%)	
Height	3,120	169 (9)	167 (10)	169 (9)	<0.001	1,312	168 (10)	167 (10)	169 (9)	<0.001
Weight	3,120	82 (19)	83 (21)	82 (19)	0.2	1,312	83 (20)	83 (21)	82 (19)	0.1
BMI	3,120	28.9 (6.0)	29.8 (6.5)	28.6 (5.8)	<0.001	1,312	29.0 (6.1)	29.8 (6.5)	28.3 (5.6)	<0.001
Smoke	3,120				<0.001	1,312				0.001
Smoke		803 (26%)	108 (16%)	695 (28%)			262 (20%)	108 (16%)	154 (23%)	
No smoke		2,317 (74%)	548 (84%)	1,769 (72%)			1,050 (80%)	548 (84%)	502 (77%)	
Drink	3,120				<0.001	1,312				0.87
Mild		2,020 (65%)	451 (69%)	1,569 (64%)			910 (69%)	451 (69%)	459 (70%)	
Moderate		811 (26%)	171 (26%)	640 (26%)			337 (26%)	171 (26%)	166 (25%)	
Heavy		289 (9.3%)	34 (5.2%)	255 (10%)			65 (5.0%)	34 (5.2%)	31 (4.7%)	
**Edu**	3,120				<0.001	1,312				0.014
Less than 9th grade		314 (10%)	36 (5.5%)	278 (11%)			99 (7.5%)	36 (5.5%)	63 (9.6%)	
9-11th grade		415 (13%)	70 (11%)	345 (14%)			141 (11%)	70 (11%)	71 (11%)	
High school		755 (24%)	140 (21%)	615 (25%)			301 (23%)	140 (21%)	161 (25%)	
AA degree		926 (30%)	206 (31%)	720 (29%)			397 (30%)	206 (31%)	191 (29%)	
College graduate		710 (23%)	204 (31%)	506 (21%)			374 (29%)	204 (31%)	170 (26%)	
PIR	3,120	2.77 (1.58)	3.05 (1.59)	2.70 (1.57)	<0.001	1,312	3.08 (1.60)	3.05 (1.59)	3.10 (1.60)	0.55
Alb (g/L)	3,120	41.7 (3.1)	41.5 (3.3)	41.8 (3.1)	0.013	1,312	41.7 (3.2)	41.5 (3.3)	41.9 (3.0)	0.005
Lymphocyte count (1,000 cells/μL)	3,120	2.04 (2.02)	2.10 (3.05)	2.02 (1.65)	0.25	1,312	2.07 (2.84)	2.10 (3.05)	2.03 (2.61)	0.7
Cr (μmol/L)	3,120	90 (43)	88 (41)	91 (43)	<0.001	1,312	89 (38)	88 (41)	90 (35)	0.001
TC (mmol/L)	3,120	4.98 (1.13)	5.01 (1.15)	4.97 (1.13)	0.52	1,312	4.96 (1.13)	5.01 (1.15)	4.91 (1.10)	0.18
Glucose (g/L)	3,120	6.07 (2.20)	5.93 (2.05)	6.10 (2.24)	0.26	1,312	6.01 (2.16)	5.93 (2.05)	6.10 (2.26)	0.58
Triglycerides (mmol/L)	3,120	1.78 (1.28)	1.74 (1.05)	1.79 (1.33)	0.55	1,312	1.74 (1.14)	1.74 (1.05)	1.73 (1.23)	0.18
Monocyte count (1,000 cells/μL)	3,120	0.60 (0.26)	0.62 (0.37)	0.60 (0.23)	0.14	1,312	0.61 (0.30)	0.62 (0.37)	0.59 (0.20)	0.055
GNRI	3,120	117 (12)	118 (13)	117 (11)	0.006	1,312	117 (12)	118 (13)	116 (11)	0.003

^1^Mean ± SD or Frequency (%).

^2^Pearson’s Chi-Squared Test; Wilcoxon Rank Sum Test.

### 3.2 Relationship between osteoarthritis and GNRI in individuals aged 60 and above

Table 2 shows the association between OA and GNRI in individuals aged 60 and above, as assessed using a multiple linear regression model. Model 1 represents the relationship between OA and GNRI in individuals aged 60 and above, without adjusting for covariates. The results indicate a positive correlation between GNRI and OA (OR: 1.015, 95% CI: 1.006–1.025, *P*-value: 0.015). When compared with the first quartile (Q1), the odds ratio (OR) for the third quartile (Q3) was 0.988 (95% CI: 0.727–1.342, *P*-value: 0.9377), while for the fourth quartile (Q4), the OR was 1.502 (95% CI: 1.104–2.047, *P*-value = 0.0098), suggesting that for each increase in GNRI from the first to the fourth quartile, the probability of OA in individuals aged 60 and above increases. Model 2, which adjusted for gender and race as covariates, shows a similar relationship between GNRI and OA. Model 3 further adjusted for alcohol consumption, PIR, lymphocyte count, creatinine, total cholesterol, triglycerides, and monocyte count. After adding these covariates, the results of both Model 2 and Model 3 were consistent with those of Model 1. Specifically, Model 2 (OR: 1.016, 95% CI: 1.007–1.026, *P* = 0.001) and Model 3 (OR: 1.017, 95% CI: 1.007–1.027, *P* = 8e-04) both showed a positive association between GNRI and OA in individuals aged 60 and above. When stratified by quartiles, the analysis revealed that, compared to Q1, the OR for Q3 in both Model 2 and Model 3 was not significant (Model 2 OR: 1.101, 95% CI: 0.803–1.509, *P* = 0.5508; Model 3 OR: 1.142, 95% CI: 0.827–1.578, *P* = 0.4199). However, for Q4, the OR was significantly higher in both models (Model 2 OR: 1.603, 95% CI: 1.168–2.204, *P* = 0.0036; Model 3 OR: 1.632, 95% CI: 1.179–2.264, *P* = 0.0032), indicating that individuals aged 60 and above with a GNRI ≥ 123.63 were associated with higher prevalence of OA. Table 3 presents the baseline characteristics of the study population (*N* = 1,312), stratified by quartiles of a continuous variable (Q1–Q4). Higher quartiles were associated with younger age (Q1: 72 ± 7 vs. Q4: 68 ± 6 years, *p* < 0.001), a higher proportion of males (Q3: 66% vs. Q1: 55%, *p* = 0.024), and increased adiposity (weight: 64 ± 11 kg to 105 ± 19 kg; BMI: 22.9 kg/m^2^ to 36.6 kg/m^2^, *p* < 0.001). Smoking frequency decreased with increasing quartiles (31-12%, *p* < 0.001). Metabolic parameters demonstrated progressive increases in glucose (5.62 ± 1.85 to 6.23 ± 2.08 mmol/L) and triglycerides (1.38 ± 1.03 to 2.01 ± 1.20 mmol/L; *p* < 0.001). Serum albumin levels peaked in Q3 (42.5 ± 2.7 g/L) compared to Q1 (40.2 ± 3.2 g/L), while lymphocyte counts followed a U-shaped distribution (Q1: 3.64 ± 2.02 × 10^9^/L; Q4: 3.87 ± 2.22 × 10^9^/L). No significant interquartile differences were observed for race, alcohol use, education, Cr, TC or Monocyte (all *p* > 0.05).

**TABLE 2:** Univariate logistic regression analysis of the association between GNRI exposure and OA.

		Model1			Model2			Model3	
**Characterisitic**	**OR**	**95%CI**	***P*-value**	**OR**	**95%CI**	***P*-value**	**OR**	**95%CI**	***P*-value**
**GNRI**									
GNRI continuous	1.015	(1.006,1.025)	0.0015	1.016	(1.007,1.026)	0.001	1.017	(1.007,1.027)	8e-04
**GNRI quantile**									
Q1 (low)	Ref	Ref		Ref	Ref		Ref	Ref	
Q2	0.782	(0.575,1.063)	0.1174	0.824	(0.601,1.129)	0.2294	0.844	(0.613,1.161)	0.2967
Q3	0.988	(0.727,1.342)	0.9377	1.101	(0.803,1.509)	0.5508	1.142	(0.827,1.578)	0.4199
Q4 (high)	1.502	(1.104,2.047)	0.0098	1.603	(1.168,2.204)	0.0036	1.632	(1.179,2.264)	0.0032
p for trend			0.0036			9e-04			8e-04

GNRI: Q1: GNRI < 109.1; Q2: 109.1 ≤ GNRI < 116.26; Q3: 116.26 ≤ GNRI < 123.63; Q4: GNRI ≥ 123.63. The model 1 was the crude model; The model 2 was adjusted by Gender, Race; The model 3 was adjusted by Gender, Race, Drink, PIR, Lymphocyte count, Cr, Total Cholesterol, Triglycerides, Monocyte count.

**TABLE 3:** Comparison of baseline characteristics across GNRI quartiles (Q1-Q4).

Variables	Total^1^	Q1	Q2	Q3	Q4	*P*-value^2^
		**[85.9-109.1]**	**[109.1-116.3]**	**[116.3-123.6]**	**[123.6-179.1]**	
		**N = 328^1^**	**N = 330^1^**	**N = 325^1^**	**N = 329^1^**	
Gender						0.024
Male	791 (60%)	179 (55%)	202 (61%)	215 (66%)	195 (59%)	
Female	521 (40%)	149 (45%)	128 (39%)	110 (34%)	134 (41%)	
Age (years)	70 (7)	72 (7)	71 (7)	70 (7)	68 (6)	<0.001
Race						0.2
Hispanic_American	102 (7.8%)	15 (4.6%)	25 (7.6%)	30 (9.2%)	32 (9.7%)	
Other_Hispanic	86 (6.6%)	15 (4.6%)	23 (7.0%)	28 (8.6%)	20 (6.1%)	
Non-Hispanic_white	850 (65%)	221 (67%)	215 (65%)	200 (62%)	214 (65%)	
Non-Hispanic_black	212 (16%)	57 (17%)	49 (15%)	54 (17%)	52 (16%)	
Other_races	62 (4.7%)	20 (6.1%)	18 (5.5%)	13 (4.0%)	11 (3.3%)	
Height (cm)	168 (10)	167 (9)	168 (10)	169 (9)	169 (10)	0.001
Weight (kg)	83 (20)	64 (11)	76 (11)	85 (11)	105 (19)	<0.001
BMI	29.0 (6.1)	22.9 (2.7)	26.8 (2.5)	29.7 (2.3)	36.6 (5.5)	<0.001
Smoke						<0.001
Smoke	262 (20%)	103 (31%)	66 (20%)	52 (16%)	41 (12%)	
No smoke	1,050 (80%)	225 (69%)	264 (80%)	273 (84%)	288 (88%)	
Drink						0.9
Mild	910 (69%)	227 (69%)	235 (71%)	226 (69%)	222 (68%)	
Moderate	337 (26%)	85 (26%)	82 (25%)	86 (26%)	84 (26%)	
Heavy	65 (5.0%)	15 (4.6%)	14 (4.2%)	15 (4.6%)	21 (6.4%)	
Edu						0.4
Less than 9th GRADE	99 (7.5%)	26 (7.9%)	28 (8.5%)	20 (6.2%)	25 (7.6%)	
9-11th grade	141 (11%)	39 (12%)	39 (12%)	29 (8.9%)	34 (10%)	
High_school	301 (23%)	80 (24%)	68 (21%)	74 (23%)	79 (24%)	
AA_degree	397 (30%)	85 (26%)	91 (28%)	114 (35%)	107 (33%)	
College_graduate	374 (29%)	98 (30%)	104 (32%)	88 (27%)	84 (26%)	
PIR	3.08 (1.60)	3.00 (1.63)	3.10 (1.55)	3.22 (1.61)	2.98 (1.59)	0.2
Alb	41.7 (3.2)	40.2 (3.2)	41.8 (3.1)	42.5 (2.7)	42.3 (3.1)	<0.001
Lymphocyte	2.84 (2.07)	3.64 (2.02)	1.96 (1.54)	2.06 (1.27)	3.87 (2.22)	<0.001
Cr	89 (38)	88 (34)	94 (58)	87 (25)	87 (25)	0.6
TC	4.96 (1.13)	4.93 (1.15)	5.06 (1.16)	4.99 (1.11)	4.85 (1.07)	0.13
Glucose	6.01 (2.16)	5.62 (1.85)	6.13 (2.56)	6.08 (2.04)	6.23 (2.08)	<0.001
Triglycerides	1.74 (1.14)	1.38 (1.03)	1.69 (1.11)	1.87 (1.14)	2.01 (1.20)	<0.001
Monocyte	0.61 (0.30)	0.62 (0.40)	0.58 (0.19)	0.59 (0.23)	0.63 (0.33)	0.11

^1^Mean ± SD or Frequency (%).

^2^Pearson’s Chi-squared test, Wilcoxon rank sum test.

### 3.3 Relationship between osteoarthritis and GNRI, and subgroup analysis of RCS curves in individuals aged 60 and above

**FIGURE 2:**
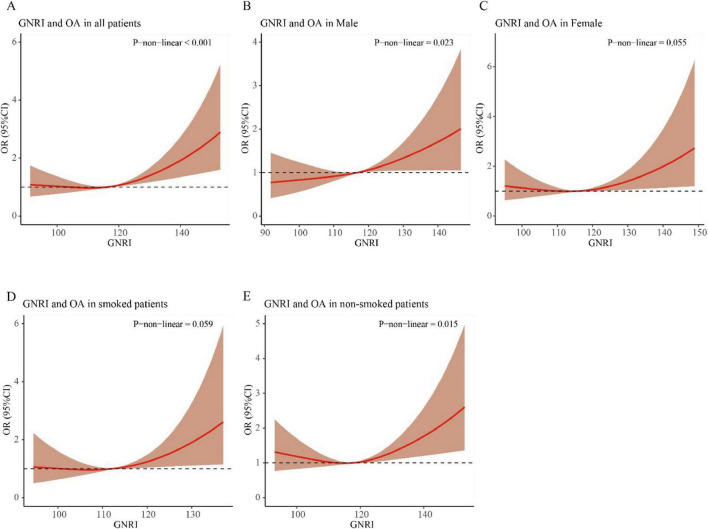
RCS curve of the association between GNRI and osteoarthritis in elderly individuals aged ≥ 60 years. **(A)** All patients; **(B)** male patients; **(C)** female patients; **(D)** smoked patients; **(E)** non-smoked patients.

RCS elucidated the relationships of GNRI with OA risk in older adults (≥ 60 years) through covariate-adjusted models. The analysis revealed a statistically significant non-linear relationship between GNRI and OA incidence in this population (P for non-linearity < 0.001, Figure 2A). Subgroup analysis by gender revealed a significant non-linear relationship between GNRI and OA incidence in the male group (P for non-linearity = 0.023, Figures 2B, C). Additionally, in the non-smoking group, a significant non-linear relationship was also observed between GNRI and the incidence of OA in individuals aged 60 and above (P for non-linearity = 0.015, Figures 2D, E).

### 3.4 Relationship between GNRI and subgroups of baseline characteristics

**FIGURE 3:**
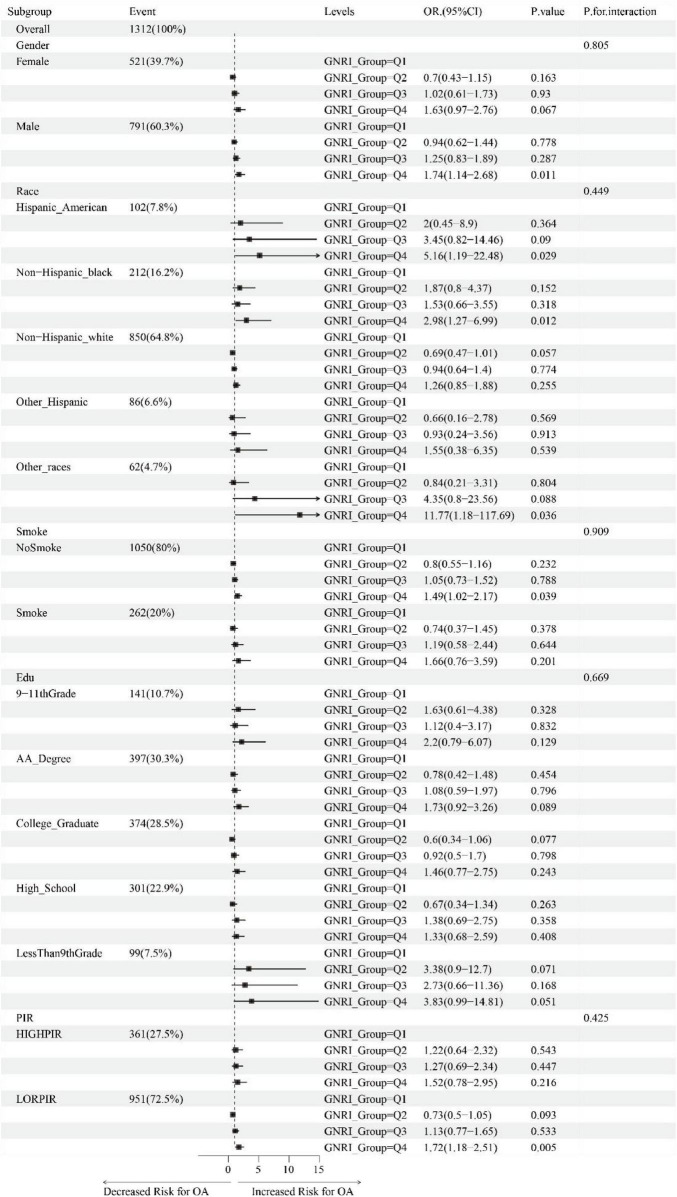
Association of GNRI with OA across baseline-characteristic subgroups (age ≥ 60).

Figure 3 illustrates the association between GNRI and OA in individuals aged 60 and above, analyzed through fully adjusted multivariable logistic regression across subgroups stratified by gender, race, smoking status, education, and PIR. We found that, within the gender subgroup, males were more sensitive to changes in GNRI (Q4 vs. Q1, OR: 1.74, 95% CI: 1.14–2.68, *P*-value: 0.011). In the race subgroups, Hispanic Americans, Non-Hispanic Black people, and other races showed higher probabilities of OA in the Q4 group compared to the Q1 group, with statistically significant differences (OR: 5.16, 95% CI: 1.19–22.48, *P*-value: 0.029; OR: 2.98, 95% CI: 1.27–6.99, *P*-value: 0.012; OR: 11.77, 95% CI: 1.18–117.69, *P*-value: 0.036). In the smoking status subgroup, non-smokers demonstrated a closer association between changes in GNRI and the occurrence of OA (Q4 vs. Q1, OR: 1.49, 95% CI: 1.02–2.17, *P*-value: 0.039). In the low PIR group, the probability of OA in individuals aged 60 and above was significantly higher in the Q4 group compared to the Q1 group (OR: 1.72, 95% CI: 1.18–2.51, *P*-value: 0.005).

### 3.5 Predictive performance comparison between GNRI and individual parameters using ROC curves

**FIGURE 4:**
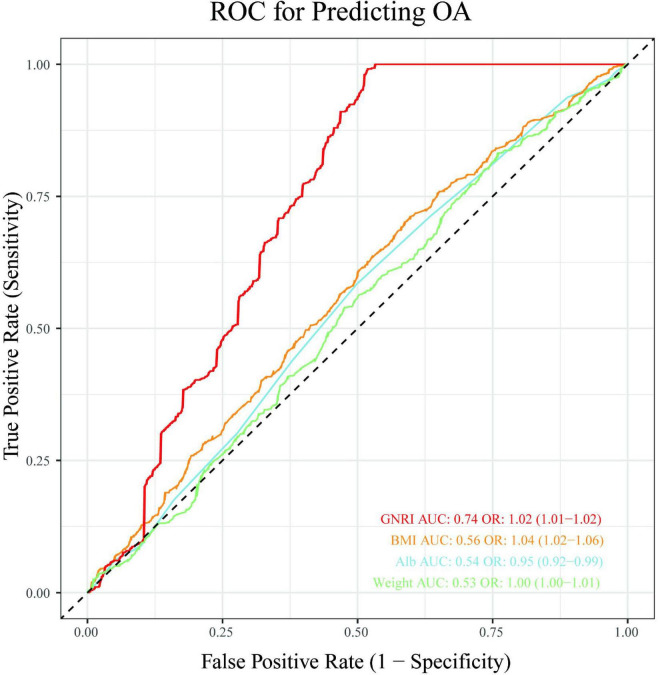
Receiver operating characteristic (ROC) analysis evaluating the predictive capacity of GNRI, BMI, albumin and weight for OA presence.

Receiver operating characteristic (ROC) analysis revealed distinct predictive capacities among the evaluated parameters (Figure 4). The GNRI demonstrated moderate discriminatory power for the presence of OA, with an area under the curve (AUC) of 0.74. In contrast, conventional anthropometric parameters exhibited limited predictive value: body mass index (BMI) (AUC = 0.56), albumin (Alb) (AUC = 0.54), and weight (AUC = 0.53).

## 4 Discussion

OA represents a major global health challenge among age-related disorders, with epidemiological projections indicating accelerated growth in disease burden concurrent with population aging trends. Identifying OA risk in older adults facilitates targeted preventive strategies and clinical interventions (1). Current studies have explored various risk factors for OA, including aging, sex, trauma, and metabolic disorders (16). GNRI, which integrates anthropometric variations with biochemical biomarkers, serves as a valuable indicator to quantify health status in aging populations (4). However, existing literature lacks robust clinical studies with sufficient sample sizes to directly establish GNRI as a predictive factor or a contributor to OA incidence. This research represents the inaugural effort to systematically evaluate the relationship between GNRI and OA prevalence in elderly individuals.

This study analyzed data from 3,120 individuals aged 60 years or older, including 656 OA cases, sourced from the NHANES database (2005–2018). The analysis conducted using logistic regression demonstrated a statistically significant positive correlation between GNRI and OA incidence across all three models. Notably, individuals with GNRI ≥ 123.63 showed a significant association with the presence of OA. RCS analysis further revealed a nonlinear relationship between GNRI and OA risk in this population, particularly pronounced among males and non-smokers. Subgroup analyses demonstrated similar nonlinear trends. These associations were consistent in a nationally representative U.S. sample and showed heightened sensitivity in specific subgroups, including males, Hispanic Americans, non-Hispanic Black people, non-smokers, and individuals with low PIR. ROC analysis further demonstrated that the GNRI exhibited moderate predictive capacity for OA presence (AUC = 0.74), highlighting its significance as a multidimensional indicator in cross-sectional risk assessment.

Nutritional risk faced by older adults are linked to various comorbid conditions, including stroke, hypertension, and cognitive impairment. A Polish study reported a negative correlation between nutritional risk and OA prevalence (24), while our results suggest that excessively high GNRI, indicating over nutrition, may increase OA risk. This association may be due to the increased mechanical load on the knee joint or fat accumulation linked to higher BMI in elderly individuals with high GNRI. Existing evidence supports a strong positive correlation between obesity and OA pathogenesis (25). For instance, severe knee OA patients often exhibit excessive visceral fat deposition (26), and Australian data indicate that obese individuals have a sevenfold higher OA prevalence (27). At the same time, obesity means an increase in visceral fat (28). Visceral adipose tissue secretes pro-inflammatory mediators, including IL-6 (29), TNF-α (30), and leptin (31), which promote cartilage degradation. These findings are consistent with our observed relationship between GNRI and OA: excess nutrition appears to promote inflammatory joint degeneration—a hypothesis supported by dietary intervention studies showing that caloric restriction can relieve OA symptoms (32). Previous research has predominantly emphasized micronutrient deficiencies (18), overlooking the emerging paradigm that both under- and over-nutrition may constitute independent OA risk dimensions. Our study extends this reconceptualization by positioning GNRI as a potential arbiter of nutritional equilibrium, where deviations in either direction could compromise musculoskeletal integrity through distinct biological mechanisms.

While females are traditionally considered high-risk for OA (16), our subgroup analysis revealed greater male susceptibility to GNRI-related OA risk, highlighting the need for sex-specific therapeutic adjustments. Although current studies suggest that current and former smokers face higher OA risks (12), our analysis found non-smokers more sensitive to GNRI-associated OA risk. This aligns with evidence that smoking-induced BMI reduction may transiently lower OA risk (33), while post-cessation weight gain exacerbates knee degeneration (34). Multivariable logistic regression analysis showed that Hispanic Americans and non-Hispanic Black people with higher GNRI scores had a greater likelihood of developing OA. Individuals with lower PIR were more sensitive to increases in GNRI. This may be related to dietary quality and structure. Studies have found that adults’ dietary quality generally improves with income, and Hispanics tend to have better dietary quality than Black and White people (35). Furthermore, the obesity rate and obesity-related indicators are significantly higher among non-Hispanic Black populations (36). Furthermore, the obesity rate and obesity-related indicators are significantly higher among non-Hispanic Black populations (37). This highlights the need for personalized nutritional interventions. These subgroups may benefit from targeted strategies to reduce the risk of OA. For example, interventions focused on weight management and physical activity could help these populations maintain muscle mass, reduce fat accumulation, and decrease joint stress.

Our findings provide critical insights for policy and research. Policymakers should integrate routine nutritional assessments into elderly health screenings and prioritize nutritional factors in updated clinical guidelines. Concurrently, community- and institution-led educational campaigns should emphasize balanced nutrition to prevent OA. However, this study has limitations. The cross-sectional design of this research constrains the capability to determine causal associations between GNRl and OA. Moreover, the use of self-reported data may lead to recall bias, warranting careful interpretation of the findings. Additionally, the NHANES dataset does not categorize OA by anatomical site (e.g., knee, elbow, or hand). Although knee OA likely predominates in our aggregated results, this lack of anatomical specificity limits insights into site-specific pathophysiology. Future multicenter longitudinal studies with extended follow-up periods should incorporate datasets that include disease-subtype differentiation details. Such data are critical for dynamically assessing nutritional status, joint symptoms, and OA progression. These investigations would clarify GNRI’s role in OA pathogenesis and inform more effective preventive strategies.

## 5 Conclusion

In conclusion, research evidence demonstrates that elevated GNRI levels are significantly associated with increased OA prevalence among older adults. These findings establish GNRI as a clinically significant risk marker for OA progression in the geriatric population.

## Data Availability

The raw data supporting the conclusions of this article will be made available by the authors, without undue reservation.
